# Supplementary figures and images for: Efficacy of wooden toy training in alleviating cognitive decline in elderly individuals with cognitive impairment: A cluster randomized controlled study
Source: PLoS One. 2024 Oct 15;19(10):e0309685. doi: 10.1371/journal.pone.0309685 (PMC11478890; doi:10.1371/journal.pone.0309685)

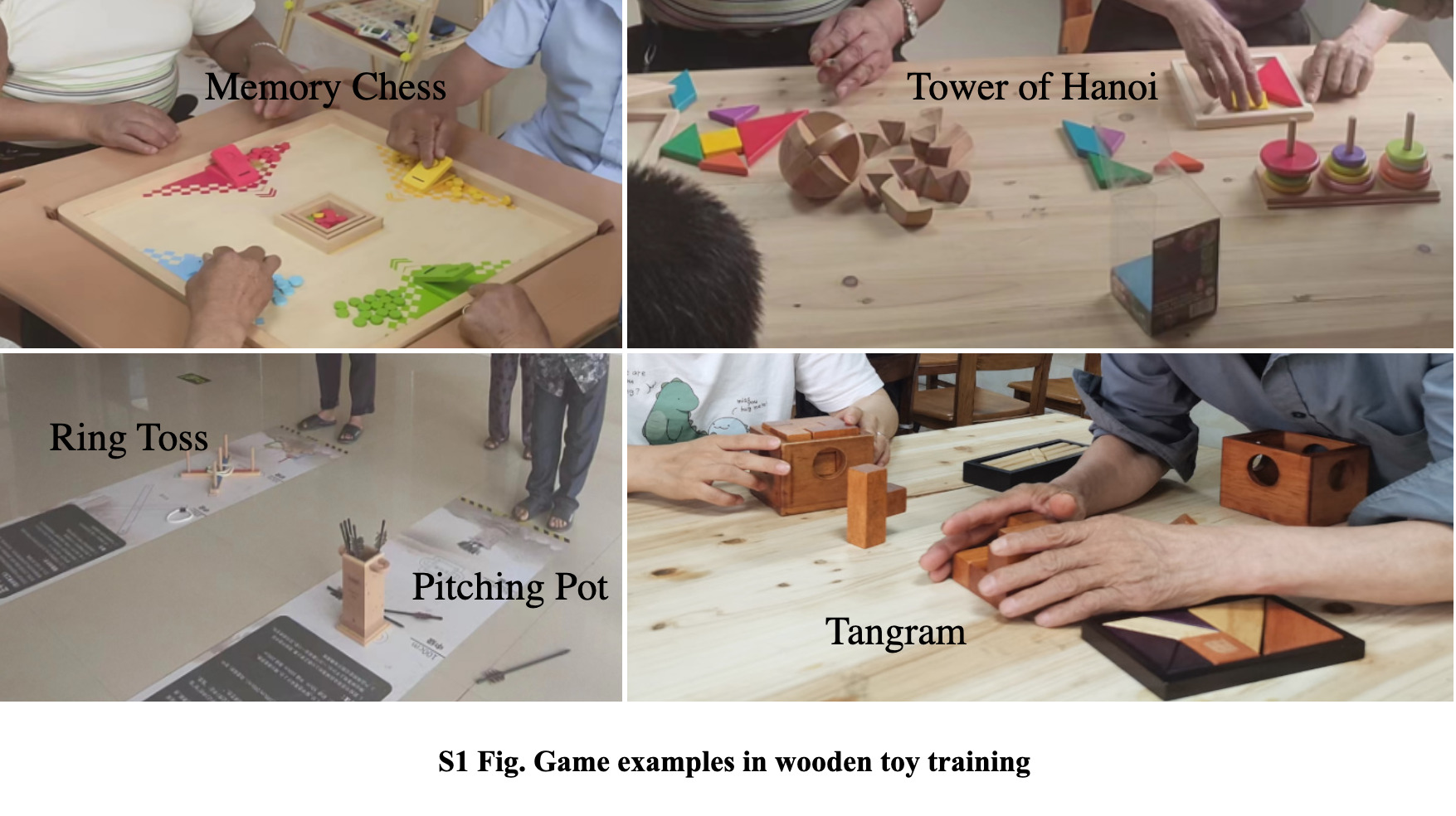

Supplement: S1 Fig — (PNG) [file pone.0309685.s001.png]

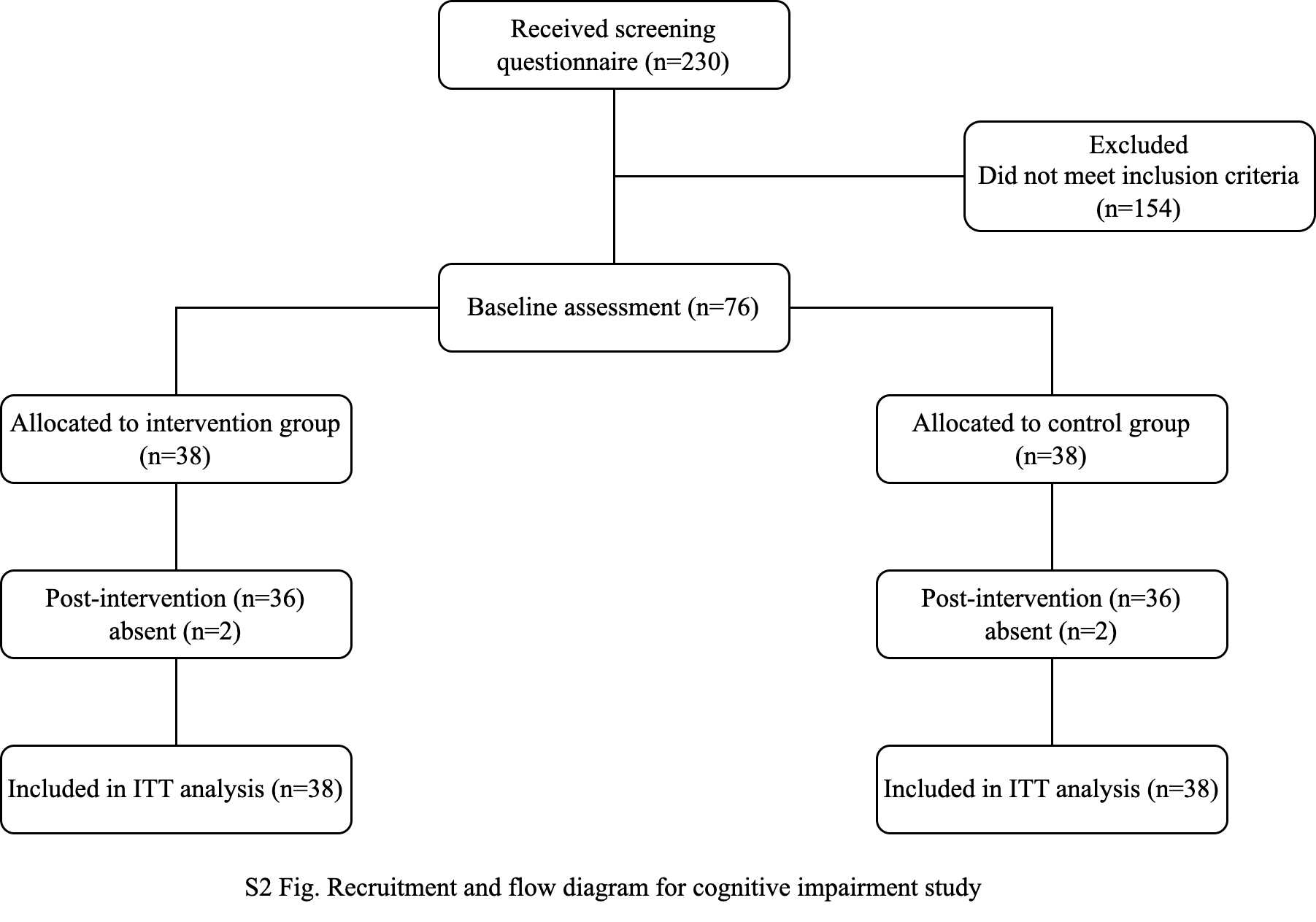

Supplement: S2 Fig — (PNG) [file pone.0309685.s002.png]
