# Supplementary material for: Efficacy of wooden toy training in alleviating cognitive decline in elderly individuals with cognitive impairment: A cluster randomized controlled study
Source: PLoS One. 2024 Oct 15;19(10):e0309685. doi: 10.1371/journal.pone.0309685 (PMC11478890; doi:10.1371/journal.pone.0309685)
Supplement: S1 Table — (DOCX) [file pone.0309685.s003.docx]

S1 Table. Distribution of MoCA item scores for intervention and control groups

| MoCA | Visualspatial/Executive | | Naming | | Attention | | Language | Abstract | | | Memory | Orientation | | |
| --- | --- | --- | --- | --- | --- | --- | --- | --- | --- | --- | --- | --- | --- | --- |
|  | Intervention group (M±SD) | Control group (M±SD) | Intervention group (M±SD) | Control group (M±SD) | Intervention group (M±SD) | Control group (M±SD) | Intervention group (M±SD) | Control group (M±SD) | Intervention group (M±SD) | Control group (M±SD) | Intervention group (M±SD) | Control group (M±SD) | Intervention group (M±SD) | Control group (M±SD) |
| Baseline | 0.92±1.17 | 1.58±1.41 | 1.82±1.01 | 2.21±0.91 | 3.37±1.65 | 3.82±2.01 | 0.87±0.99 | 1.29±1.14 | 0.18±0.39 | 0.39±0.76 | 0.24±0.63 | 0.74±1.16 | 4.71±1.21 | 4.63±1.2 |
| Postintervention | 1.05±1.37 | 1.37±1.42 | 2.45±0.69 | 2.13±1.12 | 3.58±1.67 | 3.24±2.05 | 1.24±1.13 | 1.24±1.2 | 0.39±0.68 | 0.47±0.8 | 0.82±0.93 | 0.76±1.2 | 5.76±0.54 | 4.45±0.87 |
| t value | -0.65 | 1.6 | -3.71 | 0.53 | -0.71 | 2.65 | -2.06 | 0.4 | -1.75 | -0.68 | -3.56 | -0.3 | -5.48 | 1.48 |
| p value | 0.52 | 0.12 | **0.001** | 0.6 | 0.48 | **0.012** | **0.05** | 0.69 | **0.09** | 0.5 | **0.001** | 0.77 | **0** | 0.15 |
| 95% CI | [-0.54, 0.28] | [-0.06, 0.48] | **[-0.98, -0.29]** | [-0.22, 0.38] | [-0.81, 0.39] | **[0.35, 1.29]** | **[-0.73, -0.01]** | [-0.21  ,0.32] | [-0.45,0.03] | [-0.31, 0.16] | **[-0.91,-0.25]** | [-0.21, 0.15] | **[-1.44, -0.66]** | [-0.07, 0.44] |

Abbreviations: MoCA, Montreal Cognitive Assessment.
