# Supplementary material for: Efficacy of wooden toy training in alleviating cognitive decline in elderly individuals with cognitive impairment: A cluster randomized controlled study
Source: PLoS One. 2024 Oct 15;19(10):e0309685. doi: 10.1371/journal.pone.0309685 (PMC11478890; doi:10.1371/journal.pone.0309685)
Supplement: S2 Table — (DOCX) [file pone.0309685.s004.docx]

S2 Table. Negative affective scores from baseline to postintervention

| Intervention group | fear  (m±sd) | afraid (m±sd) | unease (m±sd) | pain  (m±sd) | nervousness (m±sd) | shame  (m±sd) | guilt  (m±sd) | irritability  (m±sd) | hostility (m±sd) | fatigue (m±sd) | sluggishness (m±sd) | drowsiness (m±sd) | depression  (m±sd) | sadness  (m±sd) | grogginess (m±sd) | anxiety (m±sd) |
| --- | --- | --- | --- | --- | --- | --- | --- | --- | --- | --- | --- | --- | --- | --- | --- | --- |
| baseline | 1.37±0.13 | 1.5±0.15 | 1.89±0.21 | 1.61±0.14 | 1.58±0.15 | 1.21±0.09 | 1.16±0.07 | 1.71±0.18 | 1.32±0.11 | 2.26±0.19 | 2.18±0.19 | 2.26±0.2 | 1.79±0.15 | 1.61±0.15 | 2.03±0.22 | 1.71±0.16 |
| postintervention | 1.24±0.07 | 1.39±0.08 | 1.42±0.08 | 1.26±0.07 | 1.5±0.09 | 1.11±0.05 | 1.32±0.08 | 1.29±0.07 | 1.05±0.04 | 2.11±0.09 | 2±0.08 | 1.97±0.13 | 1.42±0.08 | 1.58±0.09 | 2.16±0.13 | 1.68±0.12 |
| t value | 0.84 | 0.66 | 2.13 | 2.18 | 0.45 | 1.16 | -1.78 | 2.3 | 2.14 | 0.85 | 0.94 | -1.4 | 2.34 | 0.15 | 0.15 | 0.16 |
| p value |  |  | 0.04 | 0.04 |  |  |  | 0.03 | 0.04 |  |  |  | 0.03 |  |  |  |
| 95% CI |  |  | [0.02, 0.92] | [0.02, 0.66] |  |  |  | [0.05, 0.79] | [0.01, 0.51] |  |  |  | [0.05, 0.69] |  |  |  |
